# CORRELATION OF NON-ALCOHOLIC FATTY LIVER DISEASE AND FEATURES OF METABOLIC SYNDROME IN MORBIDLY OBESE PATIENTS IN THE PREOPERATIVE ASSESSMENT FOR BARIATRIC SURGERY

**DOI:** 10.1590/0102-6720201600040011

**Published:** 2016

**Authors:** Fernando de BARROS, Sergio SETÚBAL, José Manoel MARTINHO, Loraine FERRAZ, Andressa GAUDÊNCIO

**Affiliations:** 1 Andarai Federal Hospital, Department of Bariatric and Metabolic Surgery, Rio de Janeiro;; 2 FIOCRUZ, Clinical Research, Rio de Janeiro;; 3 Postgraduate Program in Medical Sciences, Fluminense Federal University, Niteroi;; 4 Department of General and Specialized Surgery, Fluminense Federal University, Niteroi, RJ, Brazil.

**Keywords:** Bariatric surgery. Obesity, morbid. Fatty liver.

## Abstract

**Background:**

Obesity is an epidemic and chronic disease that can bring other comorbidities to the patient. Non-alcoholic fatty liver disease is present in up to 90% of these patients and can progress to hepatitis and hepatocarcinoma. The relationship of this liver disease and obesity is already well known; however, it is possible that some parameters of the comorbidities are more related than others in the pathophysiology of the disease.

**Aim:**

Was analyzed the relationship between non-alcoholic fatty liver disease (NAFLD) and the comorbidities of metabolic syndrome in morbidly obese patients.

**Methods:**

Was involved ultrasonography and laboratory assessment of obese patients before bariatric surgery. NAFLD was assessed using the same sonography parameters for all patients. Based on the results, the patients were divided into groups with and without NAFLD. Comparisons between them involved clinical and laboratory variables such as fasting blood glucose, insulin, HOMA-IR (homeostasis model assessment - insulin resistance), glycated hemoglobin, total cholesterol and fractions, triglycerides, alanine aminotransferase, aspartate aminotransferase, gamma glutamyl transferase, C-reactive protein, albumin and ferritin. Patients who reported alcohol abuse (defined as the consumption of >14 drinks per week) or who had hepatitis were excluded.

**Results:**

Eighty-two patients (74 women and 8 men) were studied, of whom 53 (64.6%) had NAFLD and 29 (35.4%) did not. The levels of glycated hemoglobin (p=0.05) and LDL cholesterol (p=0.01) were significantly altered in patients with NAFLD. However, weight, body mass index and excess weight did not differ significantly between the groups (p=0.835, p=0.488 and p=0.727, respectively).

**Conclusions:**

Altered LDL cholesterol and glycated hemoglobin levels were related to the presence of NAFLD.

## RESUMO

**Racional:**

A obesidade é doença epidêmica e crônica que pode trazer outras comorbidades ao paciente. A doença hepática gordurosa não alcoólica está presente em até 90% desses pacientes e pode evoluir para hepatite e hepatocarcinoma. A relação desta hepatopatia e a obesidade já é bem conhecida; porém, é possível que alguns parâmetros das comorbidades estejam mais relacionados do que outros na fisiopatogenia da doença.

**Objetivo:**

Correlacionar a doença hepática gordurosa não alcoólica (DHGNA) com as comorbidades da síndrome metabólica em pacientes obesos mórbidos em pré-operatório de cirurgia bariátrica.

**Métodos:**

Avaliação ultrassonográfica e laboratorial de pacientes obesos em pré-operatório para cirurgia bariátrica. Durante o preparo para a operação em todos os pacientes foi avaliada DHGNA através de ultrassonografia. De acordo com o resultado, os pacientes foram separados em dois grupos: sem DHGNA e com DHGNA. Para análise entre os grupos, avaliaram-se as seguintes variáveis clínicas e laboratoriais: insulina, HOMA-IR, hemoglobina glicada, colesterol total e frações, triglicerídeos, transaminase pirúvica, transaminase glutâmico oxalacética, gama glutamil transferase, proteína C reativa, albumina, ferritina. Os pacientes que relataram uso de bebida alcoólica ou que apresentaram hepatite foram excluídos do estudo.

**Resultados:**

Avaliou-se um total de 82 pacientes (74 mulheres e 8 homens), sendo 53 (64.6%) com DHGNA e 29 (35.4%) sem. Os níveis de hemoglobina glicada (p=0.05) e de LDL (p=0.01) mostraram-se mais relacionados no grupo de pacientes com DHGNA.

**Conclusão:**

A hemoglobina glicada e o LDL tiveram relação com a presença de DHGA.

## INTRODUCTION

Morbid obesity and the metabolic syndrome currently reach an expressive number of individuals worldwide and also in Brazil^8^ ^,^ ^11^. The number of bariatric surgeries in Brazil accompanies this epidemic with the accomplishment of 80,000 operations in 2014, as shown in a recent study by Brazilian Society of Bariatric and Metabolic Surgery^26^.

The comorbidities associated with morbid obesity, such as hypertension, dyslipidemia and diabetes mellitus type II, are just as serious as the primary condition itself. However, most related comorbidities go unnoticed for years before the first appearance of symptoms, such as non-alcoholic fatty liver disease (NAFLD). In the future, NAFLD may be the most common form of chronic hepatitis in the United States of America and may well replace hepatitis C as the main indication for liver transplantation^6^ ^,^ ^20^.

NAFLD is very common in the metabolic and obese population but is under-diagnosed and in routine medical practice has not received the recognition it deserves as a very severe condition. Such aggression can be observed in even young patients, sometimes requiring even early liver transplantation^25^.

NAFLD occurs in up to 20% of the general population and possibly up to 90% in obese patients^5^. NAFLD presents a wide spectrum of lesions that include steatosis, hepatitis and fibrosis. These three types of lesions can occur in different grades in the same patient. In the presence of fibrosis, progression to cirrhosis and hepatocellular carcinoma may occur in 20% and 10% of cases, respectively^1^. In a report in which biopsies were used to investigate cryptogenic cirrhosis, NAFLD was related to type 2 diabetes mellitus (T2DM) and/or obesity in 73% of cases^4^. Several factors involved have been linked to the hepatic damage, including type 2 diabetes mellitus (T2DM), insulin resistance, dyslipidemia and hypertension^10^ ^,^ ^13^ ^,^ ^19^ ^,^ ^34^.

All patients scheduled for bariatric surgery have liver evaluation tests, but unfortunately diabetes patients don't. According to some authors a liver biopsy, done before or during bariatric surgery may be required as well in cases of active or advanced liver disease^32^. The use of ultrasonography as the sole method for evaluating NAFLD has proven to be effective in the diagnosis of this condition^24^. However, there are some controversies about the physiopathological relationship between the obesity-related disorders and NAFLD. Bariatric surgery is currently considered the gold standard for weight loss treatment and the resolution of comorbidities, as well as the accepted treatment for NAFLD in obese patients^14^ ^,^ ^27^ ^,^ ^31^.

The aim of this study was to investigate the relationship between NAFLD and features of morbidly obese patients during the preoperative assessment for bariatric surgery. 

## METHODS

All patients were in the preoperative assessment for bariatric surgery in the Department of Bariatric and Metabolic Surgery at Andaraí Federal Hospital. The criteria for inclusion were: morbid obesity grade II or grade III (BMI>35) in preparation for bariatric surgery. The criteria for exclusion were: age <18 years or >65 years old, chronic diseases (heart failure, chronic liver disease), and alcohol abuse (abuse was defined as the consumption of >14 drinks per week). 

### Clinical evaluation

The ultrasonography assessment was done a week before surgery, with the same measurement criteria being applied to all patients. The same surgeon recorded all anthropometric variables, namely, weight, height (measured with a Welmy^(r)^ balance) and body mass index (BMI). The ideal weight was calculated using the Lorentz formula: (height - 100) - (height - 150)/K, where K is 4 for men and 2 for women^16^. The excess weight-loss was calculated as the total weight of the patient minus the ideal weight. 

Blood samples were collected for analysis in the week before surgery. The parameters analyzed included total cholesterol, triglycerides (TG), alanine aminotransferase (ALT), aspartate aminotransferase (AST), gamma glutamyl transpeptidase (GGT), C-reactive protein (CRP), albumin, ferritin, fasting blood glucose (FBG), glycated hemoglobin (HbA1c), insulin and the homeostasis model assessment - insulin resistance (HOMA-IR)^17^. The HOMA-IR was calculated using the formula: (FBG x 0.0555 x insulin) ÷ 22.5 (assuming normal values ​​≤2.5).

Two outcomes, normal or elevated (abnormal), were considered for the laboratory analyses. The parameters adopted for abnormal values​​ were HbA1c ≥6.5, FBG ≥100 mg/dl, cholesterol ≥200 mg/dl, TG ≥150 mg/dl, HDL <40 mg/dl for men and <50 mg/dl for women. The remaining values ​​were assumed elevated when greater than the following reference values: AST <40 U/l, ALT <56 U/l, GGT <61 U/l for men and <36 U/l for women, albumin 35-55 g/l, ferritin 10-80 μg/l and CRP <1 mg/l. 

### Statistical analysis

For descriptive analyses, continuous variables were expressed as the mean ± standard deviation (SD) together with minimum and maximum values. The chi-square test was used to compare the group of patients with or without NAFLD and their comorbidities, and the nonparametric Mann-Whitney U test was used to analyze continuous variables. A value of p<0.05 indicated significance. All analyses were done using the statistical software package SPSS IMB Statistics^(r)^, version 20.0.0.

## RESULTS

**FIGURE 1:**
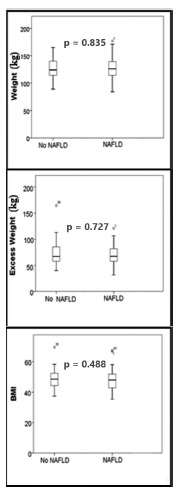
Boxplot comparisons of the weight, excess weight and body mass index (BMI) of individuals with and without non-alcoholic fatty liver disease (NAFLD). There was no significant difference in these parameters between the two groups (p = 0.835, p = 0.727 and p = 0.488 for weight, excess weight and BMI, respectively). Statistical comparisons were done using the Mann Whitney U test with p<0.05 indicating significance.

Were evaluated 82 patients, 74 women (90.2%) and 8 males (9.8%), with a mean age of 42.6±11.8 years old. Twenty-nine did not have NAFLD and 53 had some degree of liver damage (Table 1). In patients with NAFLD, the BMI was 48.2±6.8 kg/m^2^ (range: 35.3 to 57.4 kg/m^2^) and in patients without NAFLD the BMI was 49.1±7.1 kg/m^2^ (range: 37.4 to 59.6 kg/m^2^; there was no significant difference in this parameter between the two groups (p=0.488). The mean weight of patients with and without NAFLD was 128±20 kg and 126±18 kg, respectively (p=0.835). The excess weight in patients with and without NAFLD was 70.3±18.3 kg (range: 31.6 to 111.0 kg) and 74.1±25.1 kg (range: 49.0 to 115.5 kg) (p=0.727), respectively (Figure 1).

**TABLE 1:** Results for comorbidities between groups with and without NAFLD

PATIENTS		Without NAFLD (29)	NAFLD (53)	p
INSULIN	N	24 (82.8%)	35 (66.1%)	0.10
	E	5 (17.2%)	18 (33.9%)	
HOMA - IR	N	13 (44.8%)	7 (13.2%)	0.001
	E	16 (55.2%)	46 (86.8%)	
HBA1C	N	24 (82.8%)	33 (62.3%)	0.054
	E	5 (17.2%)	20 (37.7%)	
FBG	N	8 (27.6%)	18 (34.0%)	0.553
	E	21 (72.4%)	35 (66.0%)	
AST	N	29 (100%)	50 (94.3%)	0.19
	E	0 (0%)	3 (5.7%)	
ALT	N	27 (93.1%)	44 (83.0%)	0.20
	E	2 (6.7%)	9 (17.0%)	
GGT	N	22 (75.9%)	31 (58.5%)	0.11
	E	7 (24.1%)	22 (41.5%)	
LDL	N	27 (93.1%)	16 (30.2%)	0.01
	E	2 (6.9%)	37 (69.8%)	
HDL	N	6 (20.7%)	13 (24.5%)	0.69
	E	23 (79.3%)	40 (75.5%)	
CHOLESTEROL	N	14 (48.3%)	31 (58.5%)	0.37
	E	15 (51.7%)	22 (41.5%)	
TG	N	26 (89.7%)	42 (79.2%)	0.23
	E	3 (10.3%)	11 (20.8%)	
CRP	N	3 (10.3%)	6 (11.3%)	0.89
	E	26 (89.7%)	47 (88.7%)	
FERRITIN	N	25 (86.2%)	46 (86.8%)	0.94
	E	4 (13.8%)	7 (13.2%)	
ALBUMIN	N	27 (93.1%)	51 (96.2%)	0.53
	E	2 (6.9%)	2 (3.8%)	

ALT=alanine aminotransferase; AST=aspartate aminotransferase; CRP=C-reactive protein; E=elevated; FBG=fasting blood glucose; GGT=gamma glutamyl transpeptidase; HOMA-IR=homeostasis model assessment - insulin resistance; HbA1c=glycated hemoglobin; N=normal; NAFLD=non-alcoholic fatty liver disease; TG=triglycerides. P values were calculated using the chi-square test. p≤0.05 indicates significant difference.

Patients with NAFLD had a higher frequency of high levels of HbA1c (p=0.054), HOMA-IR (p=0.001) and increased levels of LDL cholesterol (p=0.01). Table 1 summarizes the results for the variables analyzed in relation to the absence or presence of NAFLD.

## DISCUSSION

For a long time, NAFLD was considered a benign condition of little clinical importance. With the worldwide epidemic of morbid obesity and metabolic syndrome, bariatric surgery and NAFLD have become the focus of intense research. Some authors go so far as to consider NAFLD as another component of the metabolic syndrome^17^. However, the pathogenesis of liver damage and obesity-related disorders is still controversial. 

Although the relationship between obesity and NAFLD is well established, but the BMI and absolute weight of the patient are apparently not directly related. Younossi et al. reported that in a significant number of non-obese patients NAFLD was not directly associated with weight (p<0.05) and concluded that these patients had a different clinical profile of obesity, but had a component of the metabolic syndrome^35^. Gupte et al. studied the relationship between T2DM and NAFLD and reported no significant differences between BMI and liver damage^12^. In our series of patients with and without NAFLD there was no significant difference between the groups in relation to patient size, i.e., absolute weight (p=0.835), BMI (p=0.488) and excess weight (p=0.727). This finding reinforced the hypothesis that the metabolic components of morbid obesity were more related to the pathogenesis of NAFLD than to patient size itself; these data may also indicate that the mechanism by which comorbidity causes NAFLD is different. Recently, the CURES study (Chennai Urban Rural Epidemiology Study) identified metabolic differences between NAFLD in obese and non-obese patients^30^. Compared with obese patients, glucose (p<0.05) and HOMA-IR (p<0.001) were lower and LDL cholesterol (p<0.001) was higher than in non-obese patients. 

NAFLD can increase transaminase levels in some patients, even in the absence of signs and symptoms. AST levels have been used as a marker for fatty deposits in the liver, and cross-sectional studies have found an association between metabolic syndrome and AST^33^. Prospective epidemiological studies have also shown that patients with NAFLD and increased AST levels have a higher risk of developing diabetes mellitus type 2^15^. Ong et al. observed that AST levels were not only an independent factor for the presence of NAFLD but also a variable associated with advanced fibrosis^23^. However, was found no difference in the AST (p=0.19), ALT (p=0.20) and GGT (p=0.11) values between obese patients with and without NAFLD. 

NAFLD and T2DM often coexist and share similar physio pathological characteristics such as an excess of adipocytes, altered metabolism and insulin resistance. NAFLD anticipates the development of T2DM and vice versa, and one condition facilitates the progress of the other^21^. In a prospective study of biopsies in patients undergoing bariatric surgery, Ong et al. found that 93% of the patients had NAFLD, and T2DM was an independent association factor. Ballestri et al. evaluated obese patients using ultrasonography and found a relationship between HOMA-IR and insulin in patients with NAFLD, except in individuals with fibrosis already established^3^. We found increased levels of HbA1c (p=0.054) and HOMA-IR (0.001) in the group with NAFLD as well. However, no association was found in this group between FBG (p=0.67) or insulin (p=0.10). We believe that FBG and insulin levels vary markedly during the day, but HbA1c is a more reliable indicator of chronic exposure to T2DM and can be a promising marker for assessing liver injury. In a cross-sectional study, Bae et al. compared the prevalence of NAFLD in relation to the severity of T2DM based on HbA1c levels and insulin resistance^2^. These authors observed, for liver injury, an odds ratios of 1.44, 7.18 and 2.62 for HbA1 levels of 5.0-5.4%, 5.5-5.9% and 6.0-6.4%, respectively, when compared to levels <4.9% (p<0.001). Based on these findings, some authors have advocated the use of hypoglycemic drugs like metformin and pioglitazone to treat NAFLD.

Dyslipidemia in patients with NAFLD has been reported in up to 80% of cases^29^. DeFilippis et al. reported that 17% of a group of patients with lipid disorders from the clinical trial MESA (Multi-Ethnic Study of Atherosclerosis) had NAFLD^7^. These authors also observed a relationship between liver damage and elevated levels of TG and LDL cholesterol (p<0.05), and low levels of HDL (p<0.05). Gupte et al. studied the degree of NAFLD and metabolic syndrome and found no significant difference in the cholesterol or triglycerides levels (p>0.05)^12^. Although the percentage of elevated LDL cholesterol levels was greater in our patients with NAFLD (p=0.01), this relationship was not observed with HDL cholesterol (p=0.69) or TG (p=0.23) levels. Based on this line of thinking, some authors advocate even medications from diabetic patients for the treatment of NAFLD, such as meftormine and apioglitazone^22^ ^,^ ^27^.

## CONCLUSION

NAFLD was closely correlated with T2DM, insulin resistance and dyslipidemia (LDL cholesterol), but was not with the size of the patient (weight, BMI and excess weight). These findings suggest that the high incidence of NAFLD in obese patients is probably related to some comorbidities. The pathophysiology of NAFLD can be multifactorial, which explains the controversy surrounding the best treatment for this condition. 
